# Lipid–Protein Nanodiscs Offer New Perspectives for Structural and Functional Studies of Water-Soluble Membrane-Active Peptides

**Published:** 2014

**Authors:** Z. O. Shenkarev, E. N. Lyukmanova, A. S. Paramonov, P. V. Panteleev, S. V. Balandin, M. A. Shulepko, K. S. Mineev, T. V. Ovchinnikova, M. P. Kirpichnikov, A. S. Arseniev

**Affiliations:** Shemyakin-Ovchinnikov Institute of Bioorganic Chemistry, Russian Academy of Sciences, Miklukho-Maklaya Str., 16/10, 117997, Moscow, Russia; Lomonosov Moscow State University, GSP-1, Leninskie Gory, 1, Bldg. 12, 119991, Moscow, Russia; Moscow Institute of Physics and Technology (State University), Institutskii Pereulok, 9, 141700, Dolgoprudny, Moscow Region, Russia

**Keywords:** antimicrobial peptides, lipid-protein nanodiscs, high-density lipoprotein particles, membrane-active peptides, membrane mimetics, neurotoxins, NMR spectroscopy

## Abstract

Lipid-protein nanodiscs (LPNs) are nanoscaled fragments of a lipid bilayer stabilized in solution by the apolipoprotein or a special membrane scaffold protein (MSP). In this work, the applicability of LPN-based membrane mimetics in the investigation of water-soluble membrane-active peptides was studied. It was shown that a pore-forming antimicrobial peptide arenicin-2 from marine lugworm (charge of +6) disintegrates LPNs containing both zwitterionic phosphatidylcholine (PC) and anionic phosphatidylglycerol (PG) lipids. In contrast, the spider toxin VSTx1 (charge of +3), a modifier of Kv channel gating, effectively binds to the LPNs containing anionic lipids (POPC/DOPG, 3 : 1) and does not cause their disruption. VSTx1 has a lower affinity to LPNs containing zwitterionic lipids (POPC), and it weakly interacts with the protein component of nanodiscs, MSP (charge of –6). The neurotoxin II (NTII, charge of +4) from cobra venom, an inhibitor of the nicotinic acetylcholine receptor, shows a comparatively low affinity to LPNs containing anionic lipids (POPC/DOPG, 3 : 1 or POPC/DOPS, 4 : 1), and it does not bind to LPNs/POPC. The obtained data show that NTII interacts with the LPN/POPC/DOPS surface in several orientations, and that the exchange process among complexes with different topologies proceeds fast on the NMR timescale. Only one of the possible NTII orientations allows for the previously proposed specific interaction between the toxin and the polar head group of phosphatidylserine from the receptor environment (Lesovoy et al., Biophys. J. 2009. V. 97. № 7. P. 2089–2097). These results indicate that LPNs can be used in structural and functional studies of water-soluble membrane-active peptides (probably except pore-forming ones) and in studies of the molecular mechanisms of peptide-membrane interaction.

## INTRODUCTION

Membrane-active peptides (MPs) are a class of biomolecules that play an important role in the existence of certain organisms and their communities. For example, antimicrobial membrane-active peptides (AMPs), which selectively act on the membranes of various cells, are among the main effectors in the “innate immunity” system, which is the earliest defense system of eukaryotes [1]. Some peptide mediators of the nervous and endocrine systems of mammalians and a number of animal toxins, targeted membrane receptors, also exhibit membrane activity and act at several stages, initially binding the membrane surrounding the receptor and only after forming a ligand-receptor complex [2, 3]. In this case, the so-called “membrane catalysis” mechanisms come into play, which greatly increases the efficiency of the ligand-receptor interaction [3].

The structural features of membrane-active peptides complicate their biophysical studies. Due to their hydrophobic properties and significant conformational mobility, many MPs form an “active” spatial structure only in the presence of a biological membrane or a suitable membrane mimetic. These factors hamper MP crystallization and necessitate the use of alternative research methods. One such method is high-resolution NMR spectroscopy, which allows one to study the spatial structure and intramolecular dynamics of MPs directly in solution of membrane mimicking media [4, 5]. The commonly used membrane mimetics have a number of drawbacks, which limit their use in the study of specific peptide–membrane interactions. For example, a large surface curvature and the loose packing of detergent-based media (in the form of micelles or small lipid-containing bicelles) can cause significant distortions in the peptide structure [6] and nonspecific peptide–detergent interactions. Meanwhile, media containing real bilayer membranes in the form of lipid vesicles or lipid/detergent bicelles have sizes that are too large for high-resolution NMR studies of MPs [4, 7].

Lipid-protein nanodiscs (LPNs) or reconstructed nascent high-density lipoprotein particles are nanosized discoid particles (typically 4 × 10 nm) containing a fragment of the bilayer lipid membrane (~150 lipid molecules), whose hydrophobic part is shielded from the solvent with two molecules of apolipoprotein or its synthetic analogue, the membrane scaffold protein (MSP) [8]. In contrast to the commonly used membrane mimetics, the membrane fragment incorporated in a lipid-protein nanodisc demonstrates increased stability and retains many biophysical properties inherent in real bilayer systems; for example the liquid crystalline to gel phase transition [9]. Recently, several papers demonstrated that LPNs can stand as alternative membrane mimetic media for structural and functional studies of membrane proteins and hydrophobic (poorly soluble) MPs [10-16]: in particular, using high-resolution NMR spectroscopy [12-15]. The use of LPNs containing various lipids and their mixtures allows one to study different functional aspects of membrane proteins and MPs [15, 16].

**Fig. 1:**
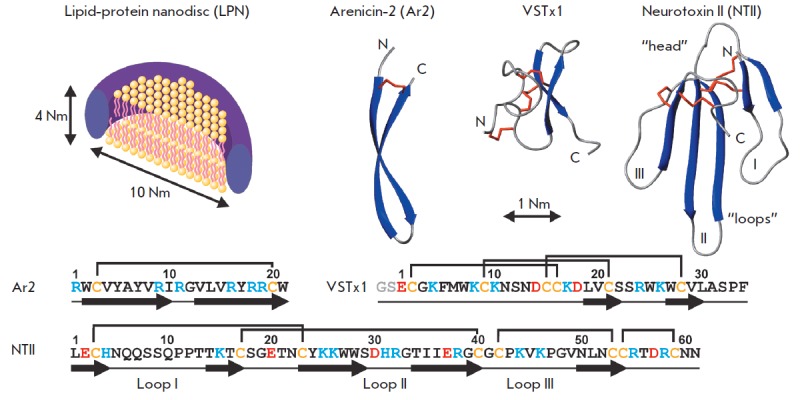
Schematic representation of a lipid-protein nanodisc and the amino acid sequences and spatial structures of arenicin-2, VSTx1, and NTII (PDB codes 2JNI, 1S6X, and 1NOR, respectively). Two MSP molecules shielding the membrane fragment of the nanodisc from the solvent are drawn as tori. Charged residues and cysteines are highlighted in the peptides sequences with color. A recombinant VSTx1 analogue with additional Gly-Ser residues at the N-terminus was used in this work (shown in gray)

The problems related to the application of LPNbased membrane mimetic media in structural and biophysical studies of water-soluble MPs have yet to be studied. It should be noted that this is far from being a trivial matter, since LPNs, unlike vesicles, bicelles, and micelles, contain an additional component, MSP, which is an anionic protein (charge of –6). In this paper, the interaction of water-soluble MPs with LPNs was studied using three model cationic β-structured peptides *(Fig. 1)*, which have different physicochemical properties and represent three classes of membrane-active compounds. The antimicrobial peptide arenicin-2 (Ar2, 21 AA, 2772 Da, charge of +6, mean Kyte-Doolittle hydrophobicity index [17] is –0.06, The maximum and minimum values of the Kyte-Doolittle hydrophobicity index [17] are +4.5 and –4.5 for poly-Ile and poly-Arg sequences, respectively.) from coelomocytes of the polychaete lugworm *Arenicola marina *interacts selectively with membranes containing negatively charged lipid molecules and forms oligomeric pores in them [18]. The VSTx1 toxin (34 AA, 4010 Da, charge of +3, hydrophobicity index is –0.27) from *Grammostola spatulata *spider venom uses the “membrane catalysis” mechanism to interact with the voltage-sensitive domains of K+-channels localized in the cell membrane, and it lacks pore-forming ability [3]. Neurotoxin II (NT II, 61 AA, 6885 Da, charge of +4, hydrophobicity index is –1.10) from *Naja oxiana *cobra venom blocks the activation of the nicotinic acetylcholine receptor through binding to its extracellular domain, but it probably also uses the “membrane catalysis” mechanism when interacting with the polar heads of phosphatidylserine (PS) from the receptor membrane environment [19]. The peptides chosen as study objects are soluble in water at millimolar concentrations; however, they differ greatly from each other in their hydrophobic/ hydrophilic properties. Thus, despite its large positive charge, Ar2 is the most hydrophobic among the studied peptides.

## EXPERIMENTAL

**LPN reconstitution and purification**

The recombinant 44–243 fragment of the human apolipoprotein A1 with a N-terminal sequence of six His residues was used as an MSP protein. The purified MSP protein, obtained as described in [20], was mixed at a molar ratio of 1 : 75 with lipids in the presence of a detergent, sodium cholate (2 : 1 cholate/lipids molar ratio), and the mixture was incubated at 4 °C for 3 h. When using saturated lipids (DLPC, DLPG), the reaction temperature was kept not lower than 25 °C. Spontaneous LPN assembly was initiated by detergent sorption to the Bio-Beads™ resin (Bio-Rad, USA) for 1.5 h. Purification of the nanodiscs was performed on a Ni^2+^ Sepharose 6 Fast Flow resin (GE Healthcare, USA) equilibrated with buffer A (20 mM Tris-HCl, 0.5 M NaCl, 1 mM NaN_3_, pH 8.0). After loading the reaction mixture, the resin was washed with a fivefold volume of buffer A. LPNs were eluted with buffer A containing 100 mM imidazole. The MSP concentration was determined spectrophotometrically by absorbance at λ = 280 nm. The LPN concentration was determined by assuming that each nanodisc contained two MSP molecules.

**Gel filtration**

was carried out on a Superdex-200 resin using a Tricorn 5/200 column (GE Healthcare, Sweden) in buffer (10 mM Tris-HCl, 0.1 M NaCl, 1 mM EDTA, 1 mM NaN_3_, pH 7.4). Thyroglobulin (669 kDa, Stokes radius R_H_ = 8.5 nm), ferritin (440 kDa, R_H_ = 6.1 nm), catalase (232 kDa, R_H_ = 5.22 nm), aldolase (158 kDa, R_H_ = 4.81 nm) BSA (67 kDa, R_H_ = 3.55 nm), and ovalbumin (43 kDa, R_H_ = 3.05 nm) were used as calibration proteins. The flow rate through the column was 0.3 ml/min. Detection was performed at 280 nm. The particle size was determined from the elution volume vs the lgR_H_ calibration curve. All the particle size (diameter) values provided below correspond to twice RH values. ;

**Preparation of a recombinant analogue of Ar2**

A recombinant arenicin-2 analogue, whose amino acid sequence is entirely consistent with that of the natural peptide, was obtained in accordance with the protocols [18, 21].

**Production and Purification of VSTx1**

The standard genetic engineering procedures were used. The *VSTx1 *gene was obtained by PCR with six overlapping synthetic oligonucleotides (Evrogen, Moscow, Russia) optimized for rare codons of* Escherichia coli*. The *VSTx1 *gene was cloned into the pET -32a(+) vector (Novagen) at the KpnI and Bam- HI sites in a single reading frame with the thioredoxin (TR X) gene. Then, the sequence encoding the enterokinase cleavage site of the fusion protein was replaced with the sequence encoding the thrombin cleavage site. The resulting plasmid was named pET /TR X-VSTx1.

BL21 (DE3) *E. coli *cells were transformed with the recombinant pET /TR X-VSTx1 vector and plated onto Petri dishes with LB agar (10 g of Bacto Tryptone, 5 g of yeast extract, 10 g of NaCl per 1 liter of the medium, pH 7.4) and ampicillin (100 mg/L). Colonies were transferred from a dish into a TB culture medium (12 g of Bacto Tryptone, 24 g of yeast extract, 4 ml of glycerol, 2.3 g of KH_2_PO_4_, 5.12 g of K_2_HPO_4_ in 1 liter of the medium, pH 7.4) containing ampicillin (100 mg/L) and cultured at 37 °C with moderate shaking until the optical density reached 0.6 o.u. The *TRX-VSTx1 *gene transcription was induced by adding isopropyl β-D-1- thiogalactopyranoside (IPTG) to a final concentration of 0.025 mM. Cultivation of the cell culture was continued in the TB medium at 37 °C overnight.

The cell culture was centrifuged (20 min, 8000 rpm, 4 °C). The cell pellet from 1 liter of the culture was resuspended in buffer A. Cells were disrupted using an ultrasonic disintegrator (Branson Digital Sonifier) for 10 s with 12-fold repetition. The lysate was centrifuged at 30,000 *g *for 30 min; the supernatant was then collected. The lysate was purified on a metal-affinity resin equilibrated with buffer A. After loading the protein sample, the column was washed with three column volumes of buffer A and three column volumes of buffer A containing 50 mM imidazole. TR XVSTx1 was eluted with buffer A containing 150 mM imidazole. After purification, specific hydrolysis of the fusion protein with thrombin was carried out. The VSTx1 sample was isolated using subtractive metal affinity chromatography. Reversed phase HPLC (C4 column, 4.6 × 250 mm, A300, Jupiter, Phenomenex) was used for the final purification of the VSTx1 sample. The toxin yield was 1 mg/L of the bacterial culture. Unlike the natural toxin, the recombinant VSTx1 analogue contained the additional N-terminal Gly-Ser residues resulting from the hydrolysis by thrombin. The identity of the recombinant toxin molecular weight to the calculated value was confirmed by mass spectrometry.

**Preparation of NTII and its ^2^H,^15^N-labeled variant**

The recombinant neurotoxin II sample was obtained according to [22]. The ^2^H,^15^N-labeled NT II sample was prepared as follows: BL21 (DE3) cells, transformed with the pET -22b(+)/STII/NT II vector [22], were plated onto Petri dishes with LB agar and ampicillin (100 mg/L). The colonies from a dish were inoculated into 10 ml of a LB medium and cultured at 37 °C for 1 h. Then, cells were added every hour with 10 ml of a LB medium prepared using deuterated water (^2^H_2_O, 99% of deuterium), until the total volume of the medium reached 110 ml. Under these conditions, cultivation was continued overnight. Afterwards, the cell pellet was aseptically harvested and re-suspended in 1 liter of a M9 minimal medium (6 g of Na_2_HPO_4_, 3 g of KH_2_PO_4_, 0.5 g of NaCl, 2 g of ^15^NH_4_Cl, 240 mg of anhydrous MgSO_4_, 11 mg of CaCl_2_, 4 ml of glycerol, 2 mg yeast extract, 200 μl of 5% thiamine chloride per 1 liter of the medium, pH 7.4) prepared with ^2^H_2_O. Cells were incubated at 37 °C until the culture optical density reached 0.6 o.u. The *stII-ntII *gene transcription was induced with IPTG, which was added to a final concentration of 0.05 mM, and the cell culture was cultured for 1 day. Isolation and purification of ^2^H,^15^N-NT II was performed according to [22].

**NMR Spectroscopy**

NMR spectra were acquired at 40–45 °C on AVANCE -700 and AVANCE -III-800 spectrometers (Bruker, Germany) equipped with cryogenically cooled triple-resonance probes at the proton resonance frequencies of 700 and 800 MHz, respectively.

To measure the isotherms of the toxins binding to LPNs and MSP molecules, the VSTx1 and NT II samples (20 mM, 10 mM Tris-Ac, pH 7.0) were titrated with solutions of nanodiscs (70 μM) of various lipid compositions or with a MSP solution (0.7 mM). The 1D 1H-NMR spectrum was acquired at each point. A data analysis was performed assuming that the intensity of the observed NMR signals of a peptide is proportional to its equilibrium concentration in solution ([P]_free_). The binding curves were fitted either to the partition equilibrium equation (1) or to the Langmuir isotherm equation (2), taking into account the dilution of the initial samples upon titration:

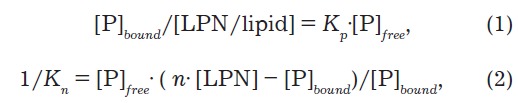



where [LPN/lipid] is the LPN concentration (assuming that one nanodisc contains two MSP molecules) or the lipid concentration (assuming that one nanodisc contains 150 lipid molecules), [P]*_bound_*is the concentration of a peptide bound to LPN ([P]*0 *= [P]*_free_*+ [P]*_bound_*), K*_p_*is the partition coefficient, *n *is the number of binding sites for a peptide on the nanodisc surface, and K*_n_*is the affinity constant of the peptide to the binding site on the nanodisc surface.

The interaction of NT II with LPN was studied using a sample containing 45 μM ^2^H,^15^N-NT II, and 75 μM LPN/POPC/DOPS (4 : 1) (10 mM Tris-Ac, pH 7.0). To identify the peptide HN-groups making contacts with the nanodisc surface, the ^1^H signal of choline group of the POPC lipid ((CH_3_)_3_N^+^_-_, chemical shift is 3.2 ppm) was saturated at a frequency of 125 Hz for 0.1, 0.3, 0.5, 0.8, 1.0, 1.5, and 3.0 s using a relaxation delay of 3 s. The changes in the intensities of the NT II cross peaks induced by presaturation of POPC choline group were observed in the 2D ^1^H,^15^N-TR OSY spectra. To identify the peptide HN-groups making contacts with the hydrophobic region of the nanodisc membrane, the paramagnetic probe of 5-doxyl-stearic acid (5-DSA) was used. 5-DSA dissolved in a small amount of methanol was added to the sample containing NT II/LPN complexes to a final concentration of 10, 30, and 75 μM. Attenuation of the NT II signal intensity arising due to the paramagnetic enhancement of the ^1^H and ^15^N nuclear relaxation was observed in the 2D ^1^H,^15^N-TR OSY spectra. The rates of the cross-correlation process between the dipole-dipole relaxation and relaxation arising from the chemical shift anisotropy of the ^15^N nucleus (η_XY_) were measured for the complexes of NT II with LPN/POPC/DOPS at 40 °C on an AVANCE -III-800 spectrometer, using amplitude modulated 2D ^1^H,^15^NTR OSY experiments [23]. The rotational correlation time (τ_R_) for the peptide HN-groups was calculated from the measured η_XY_ values.

## RESULTS AND DISCUSSION

**Interaction of arenicin-2 with nanodiscs**

The cationic AMP, Ar2, contains mainly positively charged and hydrophobic residues and has a β-hairpin structure in water, which is stabilized with one disulfide bond (*Fig. 1*) [21]. Arenicin-2 interacts selectively with membranes containing negatively charged lipid molecules and creates in them oligomeric pores, which are formed with the participation of phospholipids (the so-called “toroidal” pores) [18]. At high concentrations, Ar2 probably causes bilayer micellization [24]. As expected, the β-hairpins of individual peptides within the pore have a transmembrane orientation, so that the Nand C-terminal fragments and β-turn region come into contact with polar areas on the outer and inner sides of the membrane [18].

To study the possibility of obtaining stable Ar2–LPN complexes, nanodiscs containing neutral (POPC) and anionic (DOPG) “long-chain” lipids were used. Earlier, it had been demonstrated using CD spectroscopy that Ar2 does not interact with vesicles formed from POPC and binds with high affinity to DOPG liposomes [18, 21]. To simulate a possible transition of the Ar2 β-hairpin (length ~ 3.5 nm) in the transmembrane state, a mixture of “short-chain” lipids with different charges (DLPC/DLPG = 4 : 1, fatty acid chains with 12 carbon atoms in length, the distance between the phosphate groups on opposite sides of the membrane was ~ 3.4 vs ~ 3.7 nm for long chain lipids [25]) was used. Earlier, LPNs based on the DLPC/DLPG mixture had been used to observe the transitions between the surface-bound and transmembrane state of the channel-forming peptide antibiotic antiamoebin I [15].

**Fig. 2:**
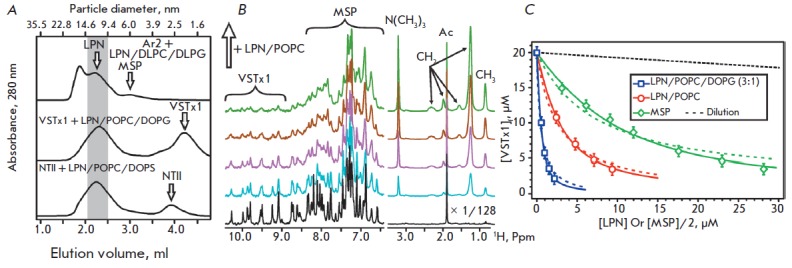
Gel filtration analysis of LPNs after MP addition and analysis of the interaction of VSTx1 with LPNs and MSP. (A). The positions of the peaks corresponding to nanodiscs, MSP aggregates, NTII, and VSTx1 are shown. (B). Interaction of VSTx1 with LPNs/POPC. The fragments of the 1D ^1^H spectra of 20 μM VSTx1 acquired at different LPN concentrations are shown. (C). The binding curves describing VSTx1 interactions with LPN and MSP are approximated by the partition equilibrium equation (eq. 1, dashed lines) and by the Langmuir isotherm (eq. 2, solid lines). Calculated parameters are summarized in the Table

During that study, an aqueous Ar2 solution was added to nanodisc samples. In all cases, even when small peptide concentrations were added, the LPN solutions were strongly opalescent, while at equimolar concentrations (Ar2/LPN = 1 : 1) and higher the solutions became opaque, which indicates nanodisc disruption and formation of larger particles. An analysis of the sample supernatants by gel filtration confirmed this assumption. Large complexes with characteristic sizes of ~ 15 nm, the residual fraction of LPNs of ~ 10–11 nm in diameter, as well as a small number of particles of ~ 6 nm in diameter were revealed in the samples (*Fig. 2A*). Comparison with the results of previous studies [20, 26] suggested that the 6 nm particles correspond to MSP aggregates. Apparently, Ar2 causes nanodisc fusion, accompanied by the release of MSP molecules. A similar process is known as high-density lipoprotein remodeling, which can occur both *in vitro *and *in vivo *upon the interaction of lipoprotein particles with lipophilic plasma proteins [27]. Previously, nanodisc fusion had been observed in cell-free protein biosynthesis systems with the cotranslational incorporation of membrane proteins into LPNs containing unsaturated lipids [26]. The spontaneous LPN fusion *in vitro *proceeds very slowly, but it could be considerably accelerated under denaturing conditions [28].

Amphiphilic MSP molecules should introduce a significant positive spontaneous curvature to the lipid bilayer for peripheral stabilization of a membrane fragment. A similar effect on the spontaneous lipid curvature is caused by a multitude of amphiphilic AMPs, whose action is mediated by the formation of “toroidal” pores, which are regions with a large positive curvature [29], or by bilayer micellization. Ar2 does not interact with POPC liposomes [21], so we may assume that the nanodisc fusion observed in the case of LPN/POPC is not directly related to the pore-forming activity of the peptide, but is caused by its attachment to a peripheral region of the LPN membrane. The attached Ar2 molecules displace certain segments of MSP, which leads to the defects in the LPN structure and induces nanodisc fusion. As a result the lipoprotein particles containing large phospholipid domains are formed and the free MSP molecules are released. In the case of LPNs containing anionic lipids, an alternative mechanism for the formation of defects in the LPN structure could be suggested as caused by the ability of Ar2 to interact directly with the nanodisc membrane.

Given that the mechanisms associated with the change in the local curvature of the lipid bilayer (“toroidal” pore formation, bilayer micellization) underlie the action of the overwhelming majority of water- soluble cationic AMPs, we can assume that many of these molecules will have a destructive effect on LPNs. Thus, nanodiscs are probably not suited as a medium for studying these peptides. It should be noted that there are other classes of channel- and pore-forming biomolecules that can be studied in LPN-based media. For example, the literature has described the formation of stable complexes of LPNs with the hydrophobic channel-former antiamoebin I (upper limit of solubility in water is 30 μM) [12, 15, 20] as well as successful incorporation of integral membrane proteins into the nanodiscs that form ion channels and pores, such as the K^+^ channel KcsA [20], nicotinic acetylcholine receptor [30], pore-forming component of the anthrax toxin [11], and a number of proteins with the β-barrel structure [14].

**Interaction of the VSTx1 toxin with nanodiscs and MSP**

The VSTx1 toxin is a small β-structured peptide stabilized by three disulfide bonds which form a “cysteine knot” (*Fig. 1*) [31]. VSTx1 weakly interacts with zwitterionic lipid membranes and has considerable affinity for the interface of phospholipid membranes, which contain anionic lipids, and yet has no membrane lytic activity [31]. According to current data, VSTx1 inhibits the voltage-dependent activation of K^+^ channels and uses “membrane catalysis” mechanisms when forming a complex with the voltage-sensing domain of the channel [3]. The toxin activity is significantly dependent on the lipid composition and mechanical state of the lipid membrane surrounding the channel [32].

Previously, a mixture of zwitterionic phosphatidylethanolamine and anionic phosphatidylglycerol lipids (POPE/POPG, 3 : 1) was used to study the interaction of VSTx1 with liposomes [3]. However, the formation of LPNs containing a significant fraction of phosphatidylethanolamine was ineffective, probably because of the high negative spontaneous curvature of the formed bilayer [20, 33]. Therefore, nanodiscs containing zwitterionic phosphatidylcholine and anionic phosphatidylglycerol (POPC and a mixture of POPC/ DOPG, 3 : 1) were used to estimate the energetics of VSTx1 interaction with LPN membranes. A MSP sample containing no lipids was used to assess the contribution of the non-specific interactions caused by the presence of a protein component in LPNs. Titration of the VSTx1 sample with a LPN solution or a solution of MSP, which forms relatively large aggregates (~ 6 nm in diameter), led to a gradual decrease in the intensity of the peptide NMR signals (*Fig. 2B*). This indicated the association of the VSTx1 molecules with the nanodisc surface or MSP. In this case, due to the slow reorientation of nanodiscs and MSP aggregates in the solution, peptide binding resulted in a significant increase in the NMR line width and a decrease in the signal intensity. Calculations demonstrated that under experimental conditions, we could safely assume that the intensity of the observed NMR signal is directly proportional to the equilibrium concentration of the free peptide in the solution ([VSTx1]_f_).

An analysis of the measured binding curves using the partition equilibrium equation (equation 1, *Fig. 2B*, *Table*) revealed that VSTx1 interacts effectively with LPNs containing anionic lipids (POPC/DOPG mixture), and that it interacts less efficiently with nanodiscs based on zwitterionic lipids (POPC). The calculated partition coefficients (*K_p_*~ 17.8 × 10^3^ and 2.6 × 10^3^ M^-1^, respectively) were much higher than the values previously observed for vesicles of POPE/POPG (3 : 1) and POPC (*K_p_*~ 2 × 10^3^ and < 0.002 × 10^3^ M^-1^, respectively) [3, 31]. These differences in the toxin affinity may be due to both the differences in the packing of phospholipid molecules in LPN membranes and vesicles [34] and the use of different buffer systems in the experiments. In papers [3, 31], the toxin binding was studied in buffers containing 150 mM KCl and NaCl, respectively, while a buffer without addition of salt was used in our work. Increased solution ionic strength, leading to partial shielding of electrostatic interactions, probably reduces the VSTx1 affinity to lipid membranes. The observed weak interaction of VSTx1 with MSP (*Fig. 2B*, *Table*), which is apparently due to the electrostatic interaction between a positively charged toxin molecule and an anionic MSP molecule, can also act as an additional factor enhancing the toxin affinity to LPNs.

**Table:** Energetic and stoichiometric parameters of VSTx1 and NTII interactions with LPNs and MSP, obtained using the partition equilibrium equation (eq. 1) and Langmuir isotherm (eq. 2)

Peptide	LPN or MSP	Partition equilibrium		Langmuir isotherm	
		K_p_(LPN, MSP×2)* ×10^6^·^M-1^	K_p_(Lipids)* ×10^3^·M^-1^	K_n_ ** ×10^6^·M^-1^	n***
VSTx1	LPN/POPC	0.39 ± 0.02	2.6 ± 0.2	0.06 ± 0.01	9.6 ± 1.5
	LPN/POPC/DOPG (3 : 1)	2.68 ± 0.24	17.8 ± 1.6	0.13 ± 0.02	34.5 ± 3.9
	MSP×2	0.10 ± 0.01		0.05 ± 0.02	3.2 ± 0.9
NT II	LPN/POPC/DOPG (3 : 1)	0.32 ± 0.01	2.13 ± 0.07		
	LPN/POPC/DOPS (4 : 1)	0.16 ± 0.01	1.07±0.07		

* K_p_ – the partition coefficient. The concentration of the “non-aqueous” phase was taken to be equal to either LPN or lipid concentrations. It was assumed that each nanodisc contains two MSP molecules and 150 lipids.

** K_n_ – the affinity constant of the peptide to the binding site on the LPN surface.

*** n – the number of binding sites on the LPN surface.

An analysis of the binding curves using the Langmuir isotherm (equation 2, *Fig. 2B*, *Table*) revealed that VSTx1 shows approximately the same affinity to the sites on the nanodisc surface or on a MSP molecule (K_n_ ~ 0.05 × 10^6^ – 0.13 × 10^6^ M^-1^, *Table*); however, the number of binding sites differs significantly. Thus, a nanodisc containing POPC (~ 150 molecules) can bind up to ~ 10 toxin molecules, and the addition of negatively charged lipids increases the number of binding sites up to ~ 35 (~ 4 lipid molecules per toxin molecule). In turn, each MSP molecule (in the absence of lipids) is able to bind up to 1.6 VSTx1 molecules, which leads to its almost complete charge compensation.

The gel filtration analysis of the VSTx1/LPN complexes revealed no nanodisc disruption upon binding of the toxin. Individual peaks can be seen on the chromatograms (*Fig. 2A*) for particles with a diameter of ~ 10–11 and ~ 2.0 nm, which probably corresponds to nanodiscs and the unbound toxin, which was dissociated from the nanodisc surface (a buffer containing 100 mM NaCl was used for the chromatography).

**Interaction of the NTII neurotoxin with nanodiscs**

**Fig. 3:**
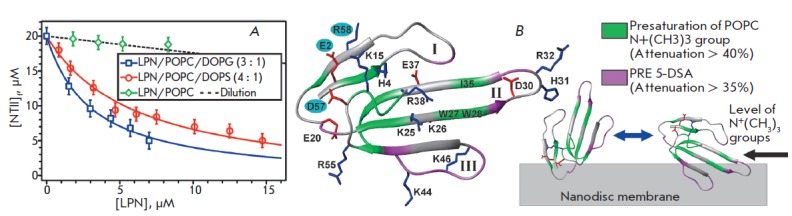
The binding curves representing NTII interactions with LPNs and the possible topology of the NTII interaction with the surface of the LPN/POPC/DOPS membrane. (A). The binding curves are approximated by the partition equilibrium equation (eq. 1). Calculated parameters are summarized in the Table. (B). The NTII ribbon is colored according to the experimental data (Fig. 4D, E). The residues forming the earlier proposed site of specific interaction with the polar headgroup of phosphatidylserine [19] are marked by blue circles

Neurotoxin II (NT II) is a cationic non-hydrophobic peptide which is stabilized by four disulfide bonds and has the so-called “three-loop” β-structure fold characteristic for snake toxins (*Fig. 1*) [35]. NT II is a highly specific inhibitor of the muscle-type nicotinic acetylcholine receptor. It blocks, with its central loop, the ligand binding sites located on the receptor extracellular domain [36]. Unlike VSTx1, NT II has no explicit membrane activity. At the same time, the ^1^H,^15^N-, and ^31^P-NMR spectroscopy investigation of NT II in the environment of DOPC/ DOPS/cholesterol (3 : 1 : 1) liposomes simulating the membrane environment of the acetylcholine receptor have suggested that the toxin action also includes elements of “membrane catalysis” mechanism [19]. Apparently, the site located in the region of the toxin “head,” near the Glu2, Asp57, and Arg58 residues (*Fig. 3B*), is able to bind, in the 1 : 1 stoichiometry, the charged headgroup of a phosphatidylserine (PS) lipid from the membrane surrounding the receptor. This interaction probably plays a role at the initial stages of NT II action and provides the toxin with the optimal orientation needed for formation of the toxin-receptor complex [19].

The LPNs assembly from a POPC/DOPS/cholesterol (3 : 1 : 1) mixture using the standard protocol for nanodisc formation (see Experimental section) appeared to be ineffective; therefore, LPNs based on a 4 : 1 POPC/DOPS mixture were used for NT II study. Furthermore, nanodiscs based on a POPC and POPC/ DOPG (3 : 1) mixture were tested for comparison. Titration of the NT II sample with nanodisc solutions revealed that the toxin does not bind to nanodiscs based on zwitterionic lipids (POPC) and shows a low affinity for LPNs containing anionic lipids (POPC/DOPG and POPC/DOPS) (*Fig. 3A*, *Table*). The higher NT II affinity for LPNs based on 3 : 1 POPC/DOPG may be explained by the larger charge of the nanodisc membrane (the relative content of a charged lipid is 25% vs 20% in the 4 : 1 POPC/DOPS membrane). Furthermore, in the membrane containing DOPS, charges of the NH_3_^+^- and COOH-groups of serine form a dipole, which may shield the negative charge of the phosphate group. Thus, the apparent charge of the polar head of DOPS will be less than that of DOPG. These findings suggest the lack of significant NT II selectivity for membranes containing different anionic lipids (phosphatidylserine and phosphatidylglycerol).

The lack of detectable binding of NT II to LPN/POPC indicates indirectly the absence of nonspecific toxin interactions with the MSP protein. As in the case of VSTx1, a gel-filtration analysis of the NT II/LPN complexes revealed no disruption of nanodiscs upon toxin binding (*Fig. 2A*). The chromatograms demonstrate the peaks corresponding to nanodiscs and the unbound toxin (~ 2.6 nm in diameter).

**Fig. 4:**
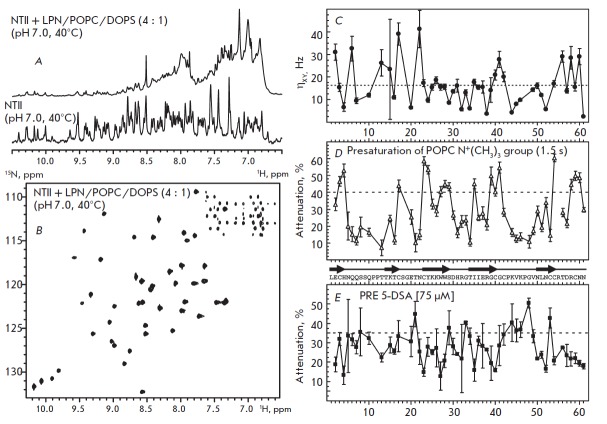
NMR analysis of NTII interaction with LPN/POPC/DOPS. (A). Comparison of the 1D ^1^H spectra of 45 μM ^2^H,^15^NNTII in water (bottom spectrum) and in complex with 75 μM LPN/POPC/DOPS (4 : 1) (upper spectrum). (B). 2D ^1^H,^15^N-TROSY spectrum of 45 μM ^2^H,^15^N-NTII in complex with 75 μM LPN/POPC/DOPS (4 : 1). (C) ^15^N cross-correlation relaxation rates (η_XY_) for NTII in complex with LPN/POPC/DOPS. Mean value is shown with dashed line. (D, E). Attenuation of cross-peak intensities in the ^1^H,^15^N-TROSY spectrum of NTII in complex with LPN/POPC/DOPS induced by (D) presaturation of the POPC choline group during 1.5 s, or (E) the paramagnetic relaxation enhancement (PRE) from 75 μM 5-DSA

The topology of the NT II interaction with the POPC/ DOPS membrane enclosed into LPN particles was studied using the 2H,15N-labeled toxin. NMR experiments were performed under conditions where the toxin was almost completely bound to the nanodisc surface. Despite the significant broadening and attenuation of the signals of the bound peptide (*Fig. 4A*), the use of the deuterated toxin and TR OSY experiments optimized to reduce the transverse relaxation of the 1H and 15N nuclei allowed us to obtain the 1H,15N-correlation spectrum of NT II in a complex with LPN (*Fig. 4B*). Comparison of the 1H and 15N chemical shifts of a NT II molecule in an aqueous environment and in a complex with LPN revealed no significant changes in the spatial structure of the toxin upon complex formation. The changes in chemical shifts exceeding 0.03 and 0.2 ppm, respectively, were observed only for one Arg32 residue (data not shown).

The cross-correlation rates of the ^15^N nuclear relaxation (η_XY_), measured for the HN-groups of NT II in complex with LPN (*Fig. 4B*), demonstrated a wide range of values (from 2.5 to 40 Hz, a mean value of 16.3 ± 9.2 Hz, a frequency of 800 MHz, 40 °C), which correspond to rotational correlation times (τ_R_) in the range from 2 to 31 ns with a mean of ~ 12.5 ns. The calculated mean τ_R_ value corresponds to the reorientation of a globular particle ~5.4 nm in diameter with a mass of ~ 34 kDa, which exceeds significantly the NT II molecule size but is significantly smaller than the nanodisc size. These findings reveal a large anisotropy of interactions within the NT II/LPN complex, which may be explained either by the presence of additional degrees of freedom of a toxin molecule within the complex or by the involvement of a NT II molecule in a fast (on the NMR scale) exchange process between the bound and unbound states.

The possible orientation of NT II on the nanodisc membrane was determined by measuring the magnetization transfer between the protons of lipids and the toxin HN-groups due to the nuclear Overhauser effect (NOE). The strongest response in the NMR spectra of the peptide was detected upon saturation of the signal of the choline group of POPC. A significant drop in the intensity of the 1H,15N-cross peaks was observed for residues located on two NT II regions: 1) in the toxin “head”, near the putative binding site for phosphatidylserine, and 2) in the central (second) loop, at the level of Trp27, Trp28, and Ile35 residues (*Fig. 3B, Fig. 4D*). The observed intensity decrease indicates the spatial proximity of the corresponding toxin HN-groups to the surface of the LPN bilayer.

Additionally, to determine the topology of NT II on the nanodisc surface we used a lipophilic spin probe 5-DSA. The spin label of 5-DSA embeds in the hydrophobic region of the bilayer close to the polar lipid headgroups. The maximum attenuation of HN-signal intensities, induced by the paramagnetic relaxation enhancement, was observed for the Thr21 residue of the toxin “head” and for residues of the third and second loops (*Fig. 3B, Fig. 4E*). This indicates the presence of a contact between the corresponding HN-groups and the hydrophobic region of the LPN bilayer.

The obtained data are not consistent with a single preferential orientation of a NT II molecule on the nanodisc membrane (*Fig. 3B*). Probably, the toxin interacts with the nanodisc surface in several (at least two) orientations and participates in the fast (on the NMR scale) exchange processes among complexes with different topologies. However, only one of the possible topologies (*Fig. 3B*) is “compatible” with the specific interaction of NT II with the polar head of phosphatidylserine at the putative binding site [19]. Thus, nonspecific electrostatic and hydrophobic interactions in the complex of NT II with LPN/POPC/DOPS have energy comparable with specific interactions.

It should be noted that the dynamic equilibrium among complexes with different topologies may play a certain role in the functioning of peripheral membrane proteins and membrane-active peptides. For example, a recent NMR study of a complex of the GTPase Rheb (Ras family) with LPN demonstrated that the protein has two possible orientations relative to the surface of the nanodisc membrane. Meanwhile, the population of these states changes during GTP hydrolysis [37].

## CONCLUSIONS

In the present study, the possibility of using LPNs to explore specific peptide/membrane interactions and the mechanisms of “membrane catalysis” in the functioning of membrane-active water-soluble antimicrobial peptides and neuropeptides was investigated. Three model β-structured peptides (arenicin-2, VSTx1, and NT II) were used. It was found that nanodiscs containing phosphatidylcholine and phosphatidylglycerol molecules can disintegrate upon interaction with cationic pore-forming peptides. Probably LPNs are not suited for structural and functional investigation of watersoluble pore-forming peptides. Meanwhile, the media based on LPNs can be used to study the energetics, stoichiometry, and topology of the interaction of membrane- active neurotoxins with a lipid membrane. In the course of such studies, one needs to consider the possibility of non-specific interactions of peptide molecules with the protein component (MSP) and lipid membrane of a nanodisc.

## Abbreviations

5-DSA: Description5-doxyl-stearic acid
AMP: antimicrobial membrane-active peptide
Ar2: arenicin-2 from marine polychaeta lugworm Arenicola marina
DLPC: dilauroyl phosphatidylcholinedilauroyl phosphatidylcholine
DLPG: dilauroyl phosphatidylglycerol
DOPG: dioleoyl phosphatidylglycerol
DOPS: dioleoyl phosphatidylserine
LPN: lipidprotein nanodisc
MP: membrane-active peptide
MSP: 44-243 fragment of human apolipoprotein A1 (membrane scaffold protein)
NTII: neurotoxin II from Naja oxiana cobra venom
POPC: palmitoyloleoyl phosphatidylcholine
POPE: palmitoyloleoyl phosphatidylethanolamine
POPG: palmitoyloleoyl phosphatidylglycerol
RH: hydrodynamic radius of a particle, Stokes radius
TROSY: transverse relaxation-optimized spectroscopy
TRX: thioredoxin from Escherichia coli
VSTx1: voltage sensor toxin from Grammostola spatulata spider venom
ηXY: the rate of cross-correlation between dipole-dipole and chemical shift anisotropy relaxation of the 15N nucleus
τR: effective rotational correlation time
